# Advanced multi-technique aeromagnetic and remote sensing characterization of a small-scale gold prospect: a case study from the Um Salim area

**DOI:** 10.1038/s41598-026-57415-3

**Published:** 2026-06-17

**Authors:** Mostafa Nagy, Mahmoud Hussein, Samar Boghdady, Saif M. Abo Khashaba

**Affiliations:** 1 Geology Department, Faculty of Science, Kafrelsheikh University, Kafrelsheikh, 33516 Egypt; 2 Geology Department, Faculty of Science, Al-Azhar University, Assiut Branch, 71524 Egypt; 3 Geology Department, Faculty of Science, Alexandria University, Alexandria, Egypt

**Keywords:** Machine learning, Hyperspectral data, Aeromagnetic data, Um Salim prospect, Edge-detection, Planetary science, Solid Earth sciences

## Abstract

Significant gold deposits occur within the ophiolitic rocks of the Central Eastern Desert of Egypt; yet, the structural controls governing ore distribution remain insufficiently constrained. This study applies an integrated remote sensing-aeromagnetic–structural workflow to the Um Salim area along the ENE-trending Barramiya–Um Salatit ophiolitic belt to delineate surface and subsurface architectures and assess their influence on gold localization. High-resolution lithological mapping was achieved through machine-learning classification of hyperspectral EnMap data using the Random Forest (RF) and Support Vector Machine (SVM) algorithms. SVM applied to enhanced Minimum Noise Fraction (MNF) components yielded superior performance, achieving an overall accuracy of 95.8%, a kappa coefficient of 94.6%, and an F1-score of 96.6%. Reduced-to-pole magnetic data reveal a prominent positive anomaly (~ 1400 nT) directly over the Um Salim gold mine, attributed to magnetite-bearing metavolcanics and ophiolitic serpentinite rocks. Upward continuation to 0.5–1 km confirms that this anomaly reflects a deeply rooted magnetic source. To refine structural interpretation, advanced edge-detection filters (TAHG and ILTHG) were applied, enhancing ENE–WSW, NE–SW, NW–SE, and N–S trends consistent with multi-phase deformation (D1–D3), and SAR-derived lineaments. Center for Exploration Targeting (CET)-based lineament density and orientation entropy maps delineate zones of intense structural complexity that closely correspond with mineralized shear zones and dyke-like porphyry intrusions. Depth-to-source analyses using Euler Deconvolution (EUD) and Tilt-Depth (TD) methods reveal that most magnetic bodies occur within 0–1000 m, whereas deeper (~ 1500 m) sources likely represent intrusive conduits that focused hydrothermal fluids. The novelty of this study lies in its integrated, multi-technique application of aeromagnetic analysis combined with hyperspectral machine learning to characterize a small, gold prospect and to map alteration-linked structural features at high resolution in the Barramiya–Um Salatit belt. The integrated results highlight new structurally complex zones in the eastern and southeastern Um Salim region as high-priority exploration targets, particularly along the NE–SW shear system that controls gold mineralization. This workflow provides a high-resolution, transferable strategy for targeting gold in structurally complex Precambrian terranes worldwide.

## Introduction

**Fig. 1:**
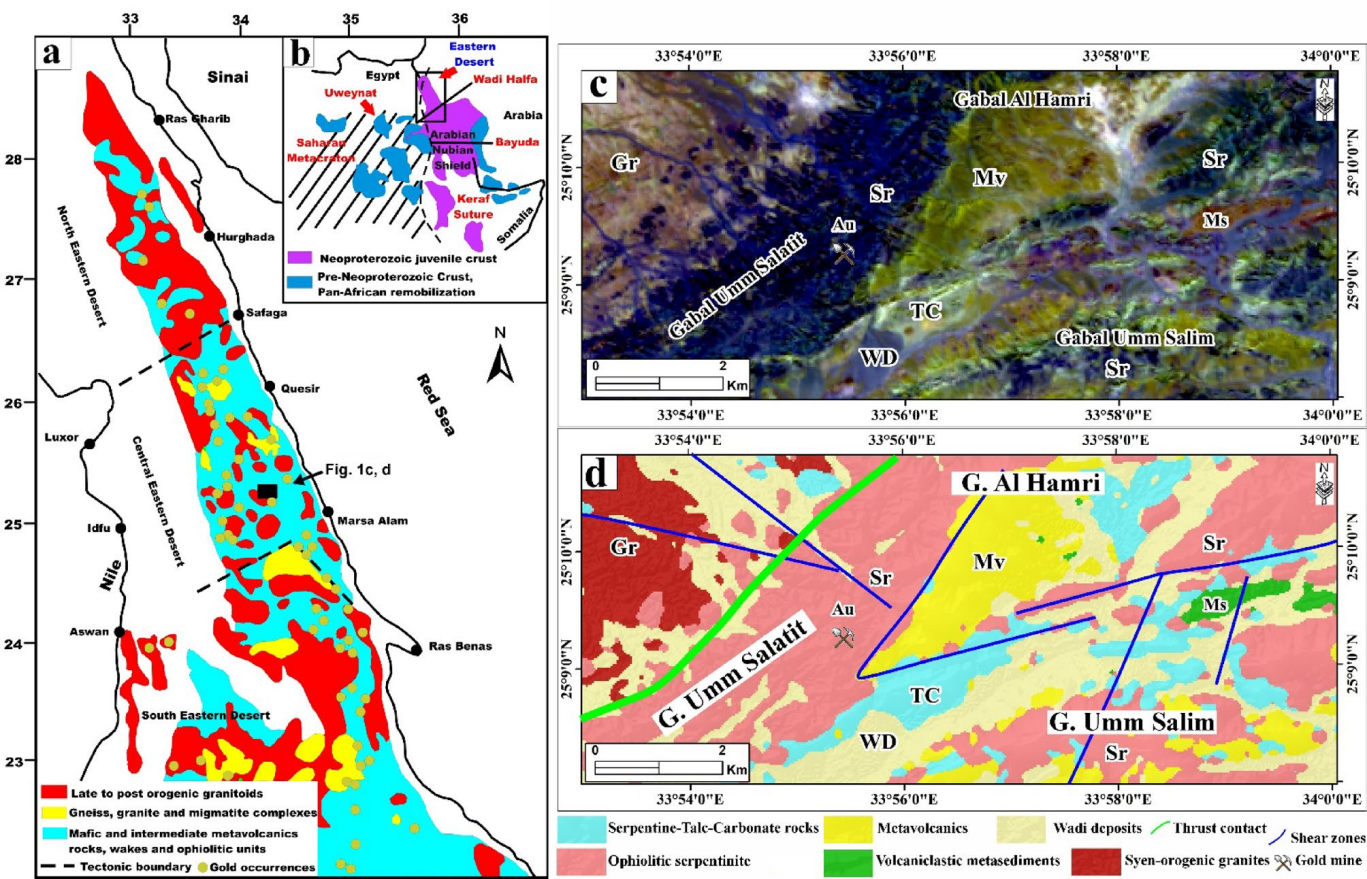
(**a**) Location map of the Arabian Nubian Shield (ANS); (**b**) Precambrian basement rocks in Egypt’s Eastern Desert are divided into NE, Central, and SE sub-provinces^43,44^; (**c**) Band combination image of EnMap data (b210, b144, b22 on RGB) of the Umm Salim area and (**d**) Geological map of the study area based on machine learning algorithm (SVM-MNFEnMAP) and previous work^11^. Created by ENVI v. 5.3 software; https://www.l3harrisgeospatial.com/Software-Technology/ENVI, which is mainly utilized for image processing and ArcGIS Desktop 10.8. https://www.esri.com/en-us/arcgis/products/arcgis-desktop/overview.

Gold, a globally significant mineral resource, has been extracted from quartz veins and alluvial deposits in eastern Egypt for over 6000 years, dating back to pre-Old Kingdom times and continuing to the present day. These mineralized terrains are part of the Arabian–Nubian Shield (ANS)^1^. Gold occurrences are widespread across the ANS (Fig. 1). Quartz–carbonate vein systems, typically hosted in metamorphic terranes and characteristic of orogenic gold environments, were the principal focus of ancient gold workings^2^. Gold at Um Salim occurs predominantly in quartz veins hosted by metavolcanics and mylonitized shear zones^3^. Gabal Um Salim represents a significant example of Eastern Desert orogenic gold systems, combining favorable lithological hosts, shear-zone structures, and hydrothermal alteration, and remains a promising site for further exploration and development.

Conventional “direct” exploration techniques, such as geological mapping, surface sampling, and drilling, provide essential information on the style and spatial occurrence of gold mineralization. In the ANS, however, the substantial thickness of Neoproterozoic successions means that these methods alone are often insufficient and must be preceded and guided by “indirect” investigations, particularly remote sensing and geophysical surveys, to refine targets prior to detailed drilling and resource evaluation. Integrated workflows that combine geological, remote sensing, and geophysical datasets have been shown to significantly improve the efficiency and cost-effectiveness of exploration targeting^4^.

Remote sensing has become a crucial tool for lithological mapping and detecting rare, precious, and radioactive mineralization, as it uses satellite imagery and spectral data to delineate distinctive mineral signatures and associated alteration zones^5–13^. Over the past decade, advances in hyperspectral imaging have transformed geological mapping by enabling pixel-level mineral identification from high-resolution spectra and greatly enhancing the detection of subtle alteration minerals critical to mineral exploration and resource evaluation. Spaceborne hyperspectral data from the EnMAP mission provide a powerful basis for ore targeting, because they resolve diagnostic VNIR–SWIR absorption features of key alteration and ore minerals at 30 m spatial resolution^11,12^. Recent studies show that EnMAP Level-2 A products can accurately map different alteration zones and other metallogenic provinces, yielding mineralogical maps that outperform those derived from conventional multispectral sensors and directly constrain fluid pathways, alteration zonation, and redox conditions during ore formation^11–13^. Additionally, machine learning algorithms such as Support Vector Machines (SVM) and Random Forest (RF) classifiers provide accurate lithological mapping using hyperspectral images such as EnMap and PRISMA^8–13^.

Using a high-resolution aeromagnetic dataset has proven highly effective in delineating geological structures and defining zones with strong potential for gold mineralization^14,15^, with notable success in terranes such as the Arabian–Nubian Shield (ANS)^16,17^. Aeromagnetic surveys have proven exceptionally valuable among geophysical techniques, particularly for gold exploration. Recent applications highlight their effectiveness in three main areas: mapping alteration zones that typically accompany gold deposits^16^, mapping structural frameworks that control ore emplacement^15^, and characterizing prospective mineralization zones^17–19^. These applications demonstrate the versatility of aeromagnetic surveys in guiding gold exploration programs^20^. Aeromagnetic surveys offer several advantages over alternative geophysical methods, notably their effectiveness in efficiently and economically mapping broad areas^21,22^. They also provide critical subsurface insights by detecting magnetic anomalies generated by ferromagnetic minerals, enabling more accurate geological interpretation^4^. This approach is particularly effective in structurally complex terrains, where conventional prospecting techniques often fail to provide reliable subsurface information^22,23^.

Despite these advantages, aeromagnetic surveys have certain limitations, especially in geologically complex regions where overlapping anomalies or intricate structures complicate interpretation^24^. However, advances in high-resolution instrumentation, enhanced data-processing techniques (e.g., edge-detection filters, Center for Exploration Targeting (CET) grid analysis, and CET porphyry methods, and integrated geological information have notably enhanced the precision of magnetic surveys, increasing their reliability in gold targeting^25,26^. Aeromagnetic surveying is increasingly used to define structural trends and hydrothermally altered belts, providing constraints that sharpen mineral prospecting strategies and reduce exploration risk^27^. In this study, aeromagnetic data were processed with advanced techniques and interpreted to resolve previously unrecognized structural fabrics and alteration patterns that govern the orogenic gold system in the Um Salim region. This work lies in (i) detailed automatic lithological map using machine learning algorithms (SVM and RF), (ii) the exclusive, multi-technique use of hyperspectral EnMap and aeromagnetic datasets to characterize a small-scale, underexplored gold prospect, (iii) the detailed quantitative mapping of surface structures using Sentinel-1 and ALOS PALSAR datasets as well as hidden structures and different alteration zones utilizing hyperspectral EnMap and aeromagnetic datasets directly linked to mineralization, and (iv) the development of a transferable aeromagnetic workflow for prospect-scale targeting. Applied to Umm Salim, this approach refines the surface and subsurface structural framework of the area and provides a practical, data-driven exploration strategy that can be readily adapted to analogous gold-bearing terranes.

## Location and geological setting

**Fig. 2:**
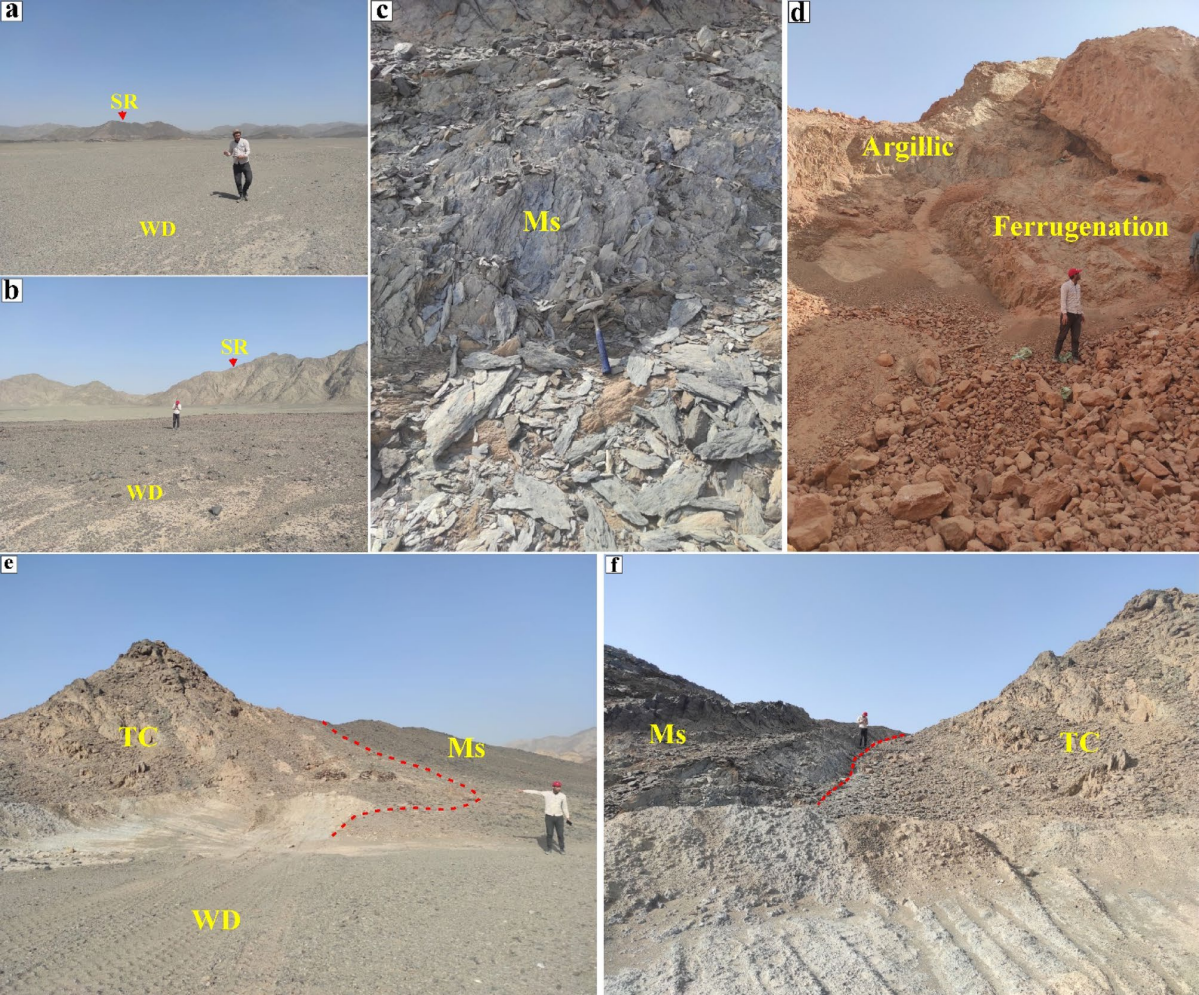
Field photographs illustrating the main lithological units and hydrothermal alteration zones of the study area. (**a**, **b**) Panoramic views of the ophiolitic serpentinite outcrops (SR) of Gabal Umm Salatit and Gabal Umm Salim, respectively. (**c**) Close-up view of the volcaniclastic metasediments (Ms) showing an intensely foliated, dark-gray, platy-to-fissile rock fabric. (**d**) Shear zone exhibiting intense hydrothermal alteration manifested as two spatially superimposed zones: an upper argillic alteration domain characterized by pale cream-to-buff clay mineral assemblages and a lower ferrugination zone dominated by vivid reddish-brown iron oxide and hydroxide minerals representing the oxidative decomposition of primary sulfide phases. The juxtaposition of argillic and ferruginous alteration within the shear corridor is consistent with multistage hydrothermal fluid circulation along a structurally controlled conduit, and constitutes a significant geochemical signature favorable for orogenic gold mineralization. (**e**, **f**) Field contact between talc-carbonate rocks (TC) and volcaniclastic metasediments (Ms). The talc-carbonate assemblage represents advanced carbonatization and talcification of the serpentinite protolith through CO₂- and SiO₂-rich hydrothermal fluid infiltration (ophicarbonation), producing a pale-greenish to cream-colored, fine-grained rock. In panel (**f**), the sharp, discordant contact between the dark Ms and the lighter TC reflects a metasomatic front driven by fluid-rock interaction along a structural boundary, with the TC zone acting as a potential geochemical trap for gold and base-metal mineralization in the broader ophiolite-hosted ore system.

**Fig. 3:**
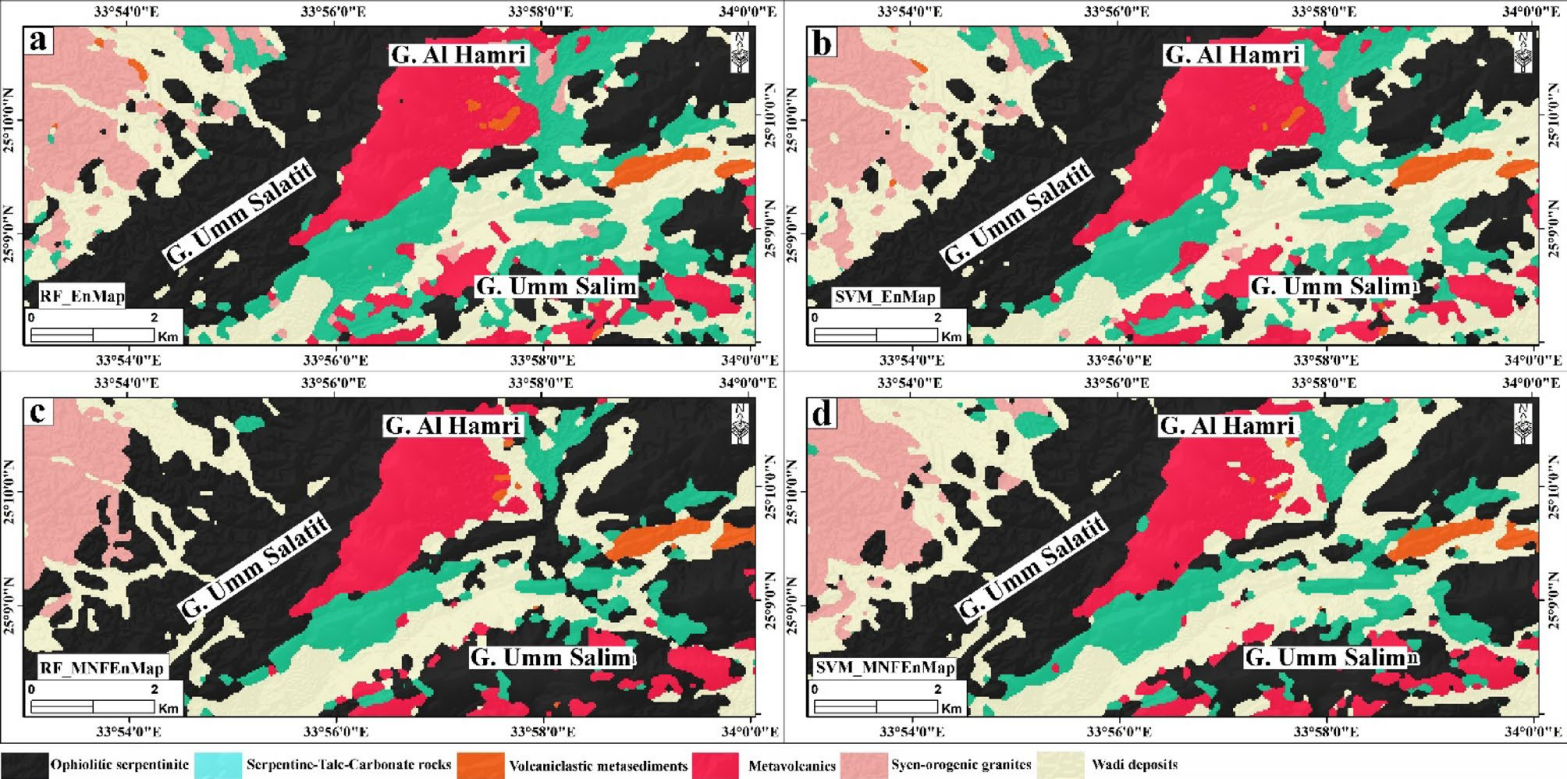
Lithological classification map generated using the Random Forest (RF) algorithm applied to the raw hyperspectral EnMap data (**a**) and enhanced Minimum Noise Fraction (MNF) transformed EnMap data (**b**). Lithological classification map generated using Support Vector Machine (SVM) algorithm applied to raw EnMap data (**c**) and enhanced Minimum Noise Fraction (MNF) transformed EnMap data (**d**). Created by QGIS Desktop 3.36.3 software; https://qgis.org/project/visual-changelogs/visualchangelog336/ and ArcGIS Desktop 10.8. https://www.esri.com/en-us/arcgis/products/arcgis-desktop/overview.

The host rocks containing the gold-hosting veins in the investigated area show remarkable diversity. They consist of intact to disrupted sections of ophiolitic sequences (including serpentinite, metagabbro, sheeted dykes, and pillow lavas) embedded in a pelitic schist matrix (Figs. 1 and 2a and b). The region also hosts an island-arc assemblage made up of metamorphosed calc-alkaline volcanic units and volcaniclastic deposits of equivalent geochemical affinity [Figs. 1, 2c and d and 3]. This succession represents a telescoped island-arc complex developed within an ensimatic ocean-basin system^28^. The complex was later thrust onto a sialic crust, which underwent varying degrees of deformation and regional metamorphism. It was subsequently overprinted by major steep shear zones and invaded by plutons of synorogenic calc-alkaline granitoids (Fig. 1d). Talc–carbonate alteration is pervasive across the ophiolitic complex, and broad zones have been mylonitized into schistose units formerly described as mudstones [Fig. 2d–f and^29^]. Mineralogically, the veins consist mainly of quartz, accompanied by minor sulphides, carbonates, micas, iron hydroxides, and occasional native gold. Pyrite is the principal sulphide mineral within the veins, followed in abundance by arsenopyrite. Additional sulphides, including pyrrhotite, sphalerite, chalcopyrite, and galena, occur in smaller amounts. In contrast, marcasite and niccolite are detected only on rare occasions^30^. Gold concentrations in ANS rocks range from 20 to 50 ppb in mafic and clastic units to nearly 200 ppb in serpentinites^31^.

Several contemporary classification systems draw upon the foundational works of^32–36^. The lithological units exposed across the study area are classified into distinct sequences, beginning with the oldest (Fig. 1). Shadli Metavolcanics form flows, sills, and thick sheets interbedded with the First Basement (Geosynclinal) Sediments^37^. The unit represents a complex assemblage of both subaerial and submarine eruptions, varying compositionally from basic through to acidic types. Serpentinites and associated ultramafic rocks are interpreted in various geological contexts. They are thought to represent ultramafic magmatic products associated with mafic volcanism and forming part of ophiolitic complexes^38^, fragments of oceanic crust obducted onto continental lithosphere^39^, or as parts of the Pan-African ophiolitic assemblages^35^. The massive serpentinite units predominantly comprise antigorite, chlorite, talc, carbonate, and tremolite minerals^3,40^.

The older granitoids consist of a group of felsic intrusive rocks that are largely intermediate in composition. Historically, these rocks have been variously termed “Grey granites”, “Synorogenic granites”^41^, and “Older granites” ^42,43^. According to^42^, their origin is commonly linked to the granitization of earlier rock units, including metasedimentary units commonly of amphibolitic character and epidioritic to metagabbroid rocks. These granitoids postdate the Geosynclinal sediments and their associated metavolcanics. The study region is covered in many places by Quaternary and wadi sediments composed of sand, pebbly detritus, and sporadic boulders. They cover the main wadis and their subsidiary tributaries. These sediments were mainly derived from the weathering of nearby rocks and accumulated throughout the Quaternary period.

## Data and methods

### Remotely sensed data

In this study, a cloud-free, atmospherically corrected hyperspectral EnMap Level 2 A dataset (ENMAP01-L2A-DT0000060680_20240206T084609Z_001_V010506_20260212T014454Z) was used to delineate lithological rock units using machine learning algorithms (SVM and RF) and to detect various hydrothermal alterations associated with gold mineralization. The EnMAP Level 2 A hyperspectral dataset was selected as the primary remote sensing data source for hydrothermal alteration mapping owing to its unparalleled spectral resolution across the VNIR–SWIR range (0.42–2.45 μm) in 242 contiguous bands at 30 m spatial resolution capabilities that are critical for resolving the specific alteration assemblages diagnostic of orogenic gold mineralization in the Umm Salim area. Unlike conventional multispectral imagery, EnMAP can discriminate clay-rich, propylitic, ferruginous, and silicified alteration types based on their diagnostic absorption features, enabling more reliable mapping of hydrothermal halos, gossans, and fluid pathways associated with ore deposition.

Sentinel-1 radar data were used for mapping surface structural lineaments and analysis of the Umm Salim area. This was obtained from the ASF Data Search Vertex https://search.asf.alaska.edu/. The LINE algorithm in PCI Geomatica (version 18) has been applied to enhanced lee PC1, generated from stacked (HV + HH) and (HV + HH) of Sentinel-1 data, to automate the extraction of structural lineaments in the study area. The LINE algorithm requires detecting several basic control constraints, such as the filter radius, edge gradient threshold, curve length threshold, and linking distance threshold. Additionally, the LINE algorithm involves three processing steps: edge detection, thresholding, and curve extraction (PCI Geomatica Manual, 2018). The extracted lineaments were used with specialized software such as ENVI 5.6, PCI-Gomatica, ArcMap, and RockWork softwares.

### Aeromagnetic data

In 1984, Aero Service collected aeromagnetic data to create a Total Magnetic Intensity (TMI) map. The data, with a geographic inclination of 41.5° North and a declination of 1.86° East, were digitized and converted into X, Y, and Z coordinates. Using Geosoft software, these coordinates were processed and gridded to produce the final TMI grid. The map was created at a scale of 1:50,000, as shown in Fig. 6. A significant step in the data processing was the application of a Fourier transform technique, following the method outlined by^45^, to the gridded TMI data. This transformation produced a reduced-to-pole (RTP) map to position magnetic anomalies over their respective sources. This enhancement clarified the magnetic signal, facilitating a more detailed assessment of structural complexity. The RTP map enhanced the clarity of the magnetic data, supporting a detailed analysis of the structural complexity in the study area.

After applying the RTP transformation, upward continuation was performed to attenuate short-wavelength magnetic anomalies, following the methodology outlined by^46^. This filtering approach offers several benefits, including reducing high-frequency noise and enhancing the detection of regional-scale magnetic features. The generated maps illustrate the distribution of magnetic susceptibility sources at different depths and highlight the most prominent geological anomalies. The primary purpose of this method is to evaluate how the depths of magnetic sources vary. Each level of upward continuation targets a specific depth range, offering a detailed representation of subsurface magnetic structures. This study applied upward continuation at 0.5 km and 1 km intervals (see Fig. 5B,C), revealing magnetic sources at depths below approximately 0.25 km and 0.5 km, respectively. These results facilitated a more precise interpretation of deeper magnetic anomalies and contributed to the structural analysis of the subsurface^47^.

#### High-precision edge detection

Edge detection techniques play a vital role in geophysical studies by helping to identify and map structural elements, including faults, lithological boundaries, dykes, and mineralization domains^17,22,23^. These methods are crucial in analyzing potential field data, as they facilitate the accurate identification of the boundaries of magnetic sources and subsurface structures^21,48^. By applying these methods, geophysicists can gain a clearer understanding of subsurface configurations, thereby enhancing the quality of geological interpretations and resource exploration.

In this study, three advanced edge detection techniques were employed to analyze the magnetic dataset, as outlined in the following sections:

*(i) Tilt Angle of the Horizontal Gradient (TAHG)*.

To enhance the effectiveness of the THG technique in delineating source boundaries^49^, proposed the Tilt Angle of the THG (TAHG), which is defined as follows:

1 $$\:TAHG=\:\:atan\:\left(\frac{{HGA}_{Z}}{\sqrt{{{HGA}_{X}}^{2}+{{HGA}_{Y}}^{2}}}\right)$$

The TAHG provides more accurate and reliable information on source boundaries than traditional methods, making it highly recommended for structural features derived from potential field datasets^50,51^.

*(ii) Improved Logistic function of total horizontal gradient (ILTHG)*.

The improved ILTHG technique introduced by^52^ refines structural interpretation by applying a logistic transformation to a vertically differentiated form of the total horizontal gradient, thereby highlighting geological boundaries more clearly in potential-field data. The method begins by evaluating the modified total horizontal gradient.

2 $${\mathrm{mTHG}}\left( {{\mathrm{x}},{\mathrm{y}}} \right) = \sqrt {\left( {\frac{{\partial D}}{{\partial {\mathrm{x}}}}} \right)^{2} + \left( {\frac{{\partial D}}{{\partial {\mathrm{y}}}}} \right)^{2} }$$

Specifically, after computing the modified total horizontal gradient (mTHG), the filter uses the first-order vertical derivative, $\:{\partial\:}_{z}\left(\mathrm{mTHG}\right)$, and normalizes it by the horizontal gradient amplitude to produce a logistic response, giving large values at structural edges. The final form of the ILTHG enhancement can be expressed as:

3 $$\:ILTHG\:=\:{\left[1+exp\left(-\frac{\frac{\partial\:mTHG}{\partial\:z}}{\sqrt{{\left(\frac{\partial\:mTHG}{\partial\:x}\right)}^{2}+{\left(\frac{\partial\:mTHG}{\partial\:y}\right)}^{2}}}\right)\right]}^{-\alpha\:}$$

Where $\:D$is the potential-field anomaly, $\:\alpha\:$is a positive scaling constant chosen by the interpreter, and $\:\partial\:/\partial\:z$denotes the first vertical derivative. The advantages of this ILTHG filter include enhanced detection of lateral edges of buried bodies, simultaneous sensitivity to both shallow and deep sources (i.e., strong and weak anomalies), suppression of false edges due to noise, and improved clarity and continuity of structural boundaries compared to classical total horizontal gradient or analytic-signal methods.

#### D-Euler depth estimation method (EUD)

Euler Deconvolution (EUD) was first proposed by^53^ as an automated technique for mapping magnetic sources and calculating their depth positions using actual magnetic profiles^54^. Later, adapted this approach to be effective with gridded magnetic data. By using spatial derivatives of potential field data, Euler deconvolution provides estimates of the depths of subsurface sources^55^. This method is widely used for mapping subsurface structural boundaries and identifying fault trends. Its core principle relies on the homogeneity constraint, which can be formulated as:

4 $$\partial {\mathrm{T/}}\partial {\text{x }}\left( {{\text{x }} - {\text{ x}}_{0} } \right) + {\text{ }}\partial {\mathrm{T/}}\partial {\text{y }}\left( {{\text{y }} - {\text{ y}}_{0} } \right) + {\text{ }}\partial {\mathrm{T/}}\partial {\text{z }}\left( {{\text{z }} - {\text{ z}}_{0} } \right) = {\text{ SI }}\left( {{\text{B }} - {\text{ T}}} \right)~$$

The geometry of subsurface magnetic bodies is expressed by the Structural Index (SI), where B denotes the background magnetic field, and T denotes the measured field at the coordinates (x, y, z). In this work, Euler deconvolution was applied to the RTP magnetic grid to reveal structural features associated with hydrothermal alteration and estimate the depths of their sources.

#### Tilt-depth method

Reference^56^ proposed a technique that uses the tilt angle (TA) to determine the depth of geological contacts. A significant advantage of this tilt-depth technique is its independence from window size (WS) and structural index (SI), enabling automated depth estimation. The mathematical formulation of the method is expressed as follows:

5 $$\:TA=\mathrm{atan}(\:\frac{h}{z}\:)$$

The TA filter was developed from the formula proposed by^57^, where *z* denotes depth, and h denotes horizontal position.

#### Center of exploration and targeting grid analysis technique (CET-GA)

The CET-GA grid analysis approach enhances magnetic image textures, making it easier to identify structurally complex areas that are valuable for exploration purposes. The structural complexity (SC) method helps detect regions with high mineral potential^58^. The CET-GA method systematically analyzes image textures to pinpoint structural lineaments, such as contacts, boundaries, and edges. The process begins with applying the standard deviation to assess magnetic variability. It then uses phase-symmetry analysis for ridge detection, followed by amplitude thresholding to extract ridge-line segments. Then, skeletonization (line thinning) is applied to generate axial lines. Finally, complexity analysis is performed to generate a density map that highlights areas with frequent contact occurrences^19,25,26^. The structural complexity (SC) method creates a junction map by identifying intersections from the detected segments. This process begins by extending each line segment indefinitely, treating the intersection points of the extended segments as potential contact locations.

#### Center of exploration and targeting porphyry analysis technique (CET-PA)

The CET Porphyry approach utilizes the Circular Feature Transform (CFT) as an initial step to detect and map circular and semi-circular anomalies^59^. Subsequently, the method identifies the centers of circular highs and lows. Utilizing the Amplitude Contrast Transform (ACT), each circular feature is represented by a distinct “halo” that highlights its outer boundary. The outputs of this process include a database file containing the positions of the detected circular features, their radial-symmetry intensities, and the optimal radius (measured in both cells and meters). Additionally, a polygon file is generated that outlines the perimeter corresponding to the highest radial symmetry around each identified center, providing a clear representation of the spatial extent of these circular structures^60^.

## Results and discussion

### Lithological discrimination using Enmap hyperspectral data

The EnMAP hyperspectral sensor, with its 242 contiguous spectral bands spanning the VNIR–SWIR range (0.42–2.45 μm) at 30 m spatial resolution, provided the spectral dimensionality required to discriminate six lithological classes across the study area: ophiolitic serpentinite (SR), metavolcanics (Mv), serpentine–talc–carbonate rocks (TC), volcaniclastic metasediments (Ms), syn-orogenic granites (Gr), and wadi deposits (WD). Two supervised machine-learning classifiers, including Random Forest (RF) and Support Vector Machine (SVM), were applied to both the raw EnMAP and the Minimum Noise Fraction (MNF)-transformed dataset (Fig. 3).

Both classifiers achieve acceptable overall accuracies on the raw EnMAP data, according to quantitative validation based on 3513 reference pixels (Tables 1 and 2). RF yields an OA of 89.84%, a Kappa coefficient of 87.00%, and a mean F1-score of 90.97% (Table 1), while SVM yields an OA = 89.38%, a Kappa coefficient of 86.41%, and a mean F1-score = 90.09% (Table 2). Relative studies demonstrate that RF manages the high dimensionality of hyperspectral feature spaces more robustly by building decorrelated decision trees that reduce overfitting^8–13^. This is consistent with RF’s slight performance advantage over SVM across all aggregate metrics on the raw data. Based on a class-level study, serpentine-talc-carbonate rocks show the best classification accuracies on the raw data (TC: F1 = 94.30% for RF, 97.88% for SVM). This is consistent with their diagnostic Mg-OH absorption at around 2.32 μm and a very uniform spectral profile. The unique feldspar and quartz spectral characteristics in the SWIR are probably what make syn-orogenic granites (Gr) always do well (F1 > 91% for both classifiers). The F1-scores for Wadi deposits (WD) were the lowest (RF: 78.45%; SVM: 78.35%). This is because they arise from mixed alluvial silt sources that include bits of all the rocks around them, which makes the spectra less clear. The biggest off-diagonal elements in the confusion matrices occur between SR and WD. The SVM misclassified 149 pixels of SR as WD, whereas the RF raw model misclassified 142 pixels of WD as SR (Tables 1 and 2).

**Table 1:** Confusion matrices and overall and class-based statistics for the RF algorithm applied to EnMap data.

Raw	SR	Mv	TC	Ms	Gr	WD	Sum	Precision%	Recall%	F1-score%
SR	1134	4	3	1	0	54	1196	94.82	88.59	91.6
MS	4	385	0	2	18	7	416	92.55	91.89	92.22
TC	0	0	248	8	1	2	259	95.75	92.88	94.3
Ms	0	5	13	565	7	36	626	90.26	92.02	91.13
Gr	0	13	2	2	280	0	297	94.28	91.5	92.87
WD	142	12	1	36	0	528	719	73.44	84.21	78.45
Sum	1280	419	267	614	306	627	3513	OA = 89.84		
Overall Accuracy%								89.84		
Kappa Accuracy%								87.00		
Mean F1 Accuracy%								90.97		

MNF	SR	Mv	TC	Ms	Gr	WD	Sum	Precision%	Recall%	F1-score%
SR	1176	0	2	0	0	18	1196	78.54	86.96	82.54
MS	17	397	0	0	0	2	416	91.49	82.76	86.91
TC	0	0	259	0	0	0	259	94.98	91.11	93
Ms	13	2	2	609	0	0	626	64.76	77.29	70.47
Gr	1	0	0	1	295	0	297	88.27	98.98	93.32
WD	99	3	0	11	2	604	719	97.76	99.95	98.84
Sum	1306	402	263	621	297	624	3513	OA = 95.08		
Overall Accuracy%								95.08		
Kappa Accuracy%								93.68		
Mean F1 Accuracy%								96.21		

**Table 2:** Confusion matrices and overall and class-based statistics for the SVM algorithm applied to EnMap data.

Raw	SR	Mv	TC	Ms	Gr	WD	Sum	Precision%	Recall%	F1-score%
SR	1108	5	5	0	0	78	1196	92.64	88.01	90.26
MS	2	374	0	0	24	16	416	89.90	93.5	91.67
TC	0	0	254	2	1	2	259	98.07	97.69	97.88
Ms	0	0	1	610	6	9	626	97.44	94.43	95.91
Gr	0	17	0	0	278	2	297	93.60	89.97	91.75
WD	149	4	0	34	0	532	719	73.99	83.26	78.35
Sum	1259	400	260	646	309	639	3513	OA = 89.84		
Overall Accuracy%								89.84		
Kappa Accuracy%								87.00		
Mean F1 Accuracy%								90.97		

MNF	SR	Mv	TC	Ms	Gr	WD	Sum	Precision%	Recall%	F1-score%
SR	1189	0	4	0	0	3	1196	99.41	92.6	95.89
MS	1	410	0	0	0	5	416	98.56	98.8	98.68
TC	0	0	259	0	0	0	259	100.00	98.48	99.23
Ms	10	3	0	603	0	10	626	96.33	96.17	96.25
Gr	0	2	0	0	295	0	297	99.33	98.99	99.16
WD	84	0	0	24	3	608	719	84.56	97.12	90.41
Sum	1284	415	263	627	298	626	3513	OA = 95.08		
Overall Accuracy%								95.76		
Kappa Accuracy%								94.57		
Mean F1 Accuracy%								96.60		

The MNF transformation applied before classification significantly improved the performance of both classifiers (Fig. 3b,d; Tables 1 and 2). RF-MNF acquired an overall accuracy (OA) of 95.08%, a Kappa statistic of 93.68%, and a mean F1 score of 96.21%. In contrast, SVM-MNF attained an OA of 95.76%, a Kappa of 94.57%, and a mean F1 of 96.60%, indicating improvements of around 5–6% points in OA compared to the raw-data models (Tables 1 and 2). The MNF rotation divides the data into signal-dominated and noise-dominated components, efficiently separating coherent spectral information from sensor noise, thereby improving inter-class separability and mitigating the Hughes phenomenon in high-dimensional feature spaces. Significantly, SVM-MNF slightly exceeds RF-MNF in all aggregate metrics, indicating that after the elimination of correlated noise, the SVM radial basis function kernel utilizes the enhanced spectral structure more efficiently than the ensemble-averaged decision boundaries of RF^7,8,13^. The MNF-based models demonstrate significant enhancements for previously challenging classes at the class level. True classification precision increases to 100.00% under SVM-MNF (up from 98.07% raw SVM), indicating flawless producer’s accuracy following noise suppression that improves the 2.32 μm Mg-OH feature. Gr achieves an F1 score of 99.16% (SVM-MNF), signifying nearly total differentiation of granite from adjacent lithologies. The WD class demonstrates significant advantages: SVM-MNF F1-score = 90.41% compared to 78.35% (raw SVM), reflecting an improvement exceeding 12% points, so validating that much of the previous ambiguity was attributable to noise rather than inherent spectral overlap. Within the RF-MNF model, metasediments (Ms) exhibit a lower F1-score of 70.47% compared to the raw RF’s 91.13%, a trend that warrants consideration. This unusual decrease may indicate an excessive reduction of spectral dimensions that encompass distinguishing characteristics of volcaniclastic metasediments, whose mineral composition (feldspar–chlorite–sericite mixtures) occupies an intermediate spectral position between metavolcanics and altered serpentinites.

Spatial comparison of the four classification maps (Fig. 3a–d) confirms that MNF-based classifications produce more geologically coherent boundaries. The SR belt along G. Umm Salatit is mapped as a continuous, well-defined unit in both MNF models. In contrast, the raw classifications yield a speckled pattern with frequent SR–WD and SR–TC misclassifications at the pixel scale. The TC class, mapped as discrete lenticular bodies within the ophiolite mélange, matches the expected field distribution of talc–carbonate pods that form through CO₂-metasomatism of serpentinite during carbonation reactions along shear zones. Misclassification between SR and TC remains the most geologically significant confusion, as both rock types share serpentine-group minerals and Mg-OH spectral absorptions; the key discriminating feature is the additional carbonate (CO₃) absorption near 2.33–2.35 μm in TC, which is resolvable by EnMAP’s narrow SWIR bands but may be masked by noise in the raw data. The metavolcanic (MS) class exhibits its clearest expression in the central G. Al Hamri area, where massive basaltic andesite flows crop out with minimal alteration, producing strong Fe²⁺ crystal-field absorptions near 1.0 μm and Fe³⁺ charge-transfer features in the VNIR that are spectrally distinct from the surrounding units.

### Hydrothermal alteration mapping

#### Alteration index distributions

**Fig. 4:**
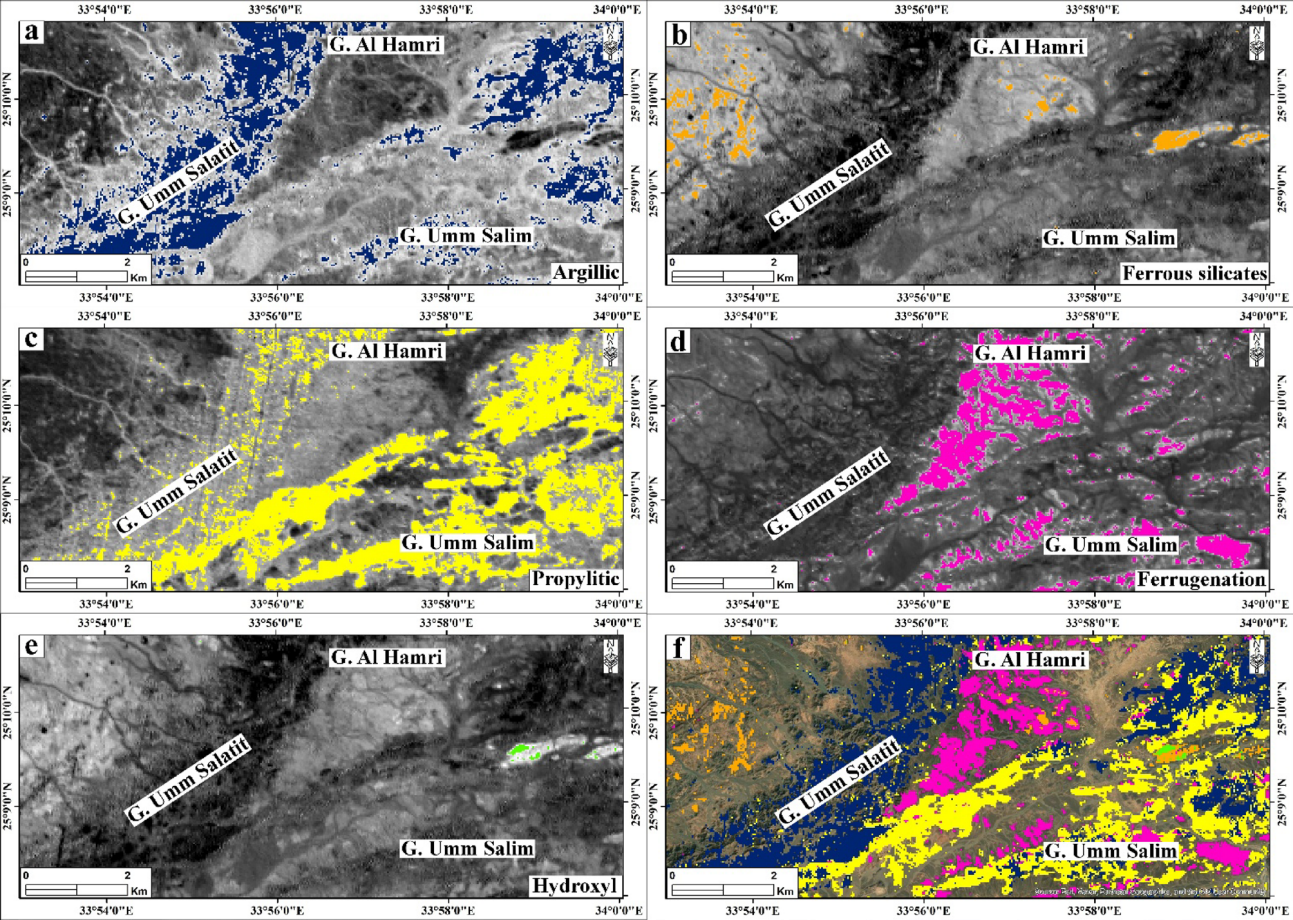
Hyperspectral EnMap alteration and mineral indices in the Umm Salim area. (**a**) Argillic alteration index (b144 + b186) / b182, emphasizing alteration zones enriched in alunite, kaolinite, and pyrophyllite. (**b**) EnMap index (b182/b144) emphasizing the ferrous silicate (biotite, chlorite, amphibole) alteration zones. (**c**) Propylitic alteration index ((b192 + b208)/b200) highlights the alteration zones enriched in chlorite, epidote, and carbonates. (**d**) Ferrugination index ((b144/b42)+(b42/b22)) emphasizes the distinct alteration zones rich in hematite and goethite. (**e**) EnMap index (b192/b186) × (b144/b186) emphasizes the hydroxyl-bearing alteration zones. (**f**) EnMap mineral indices, showing the distribution of (argillic, ferrous silicates, propylitic, ferrugination, and hydroxyl group) in the study area over a Google Earth image. Created by ENVI v. 5.3 software; https://www.l3harrisgeospatial.com/Software-Technology/ENVI, which is mainly utilized for image processing and ArcGIS Desktop 10.8. https://www.esri.com/en-us/arcgis/products/arcgis-desktop/overview.

Hyperspectral EnMap satellite data enable the assessment of the mineral composition of Earth’s materials^12^. This study utilizes EnMap mineral indices to identify alteration zones associated with gold mineralization in the Umm Salim area. Five hydrothermal alteration mineral indices, including argillic, propylitic, ferrugenation, ferrous silicates, and hydroxyl, were derived from the EnMAP VNIR–SWIR data and mapped across the study area (Fig. 4).

Argillic alteration (Fig. 4a) is characterized by intense Al-OH absorption near 2.16–2.20 μm, diagnostic of kaolinite, montmorillonite, and illite assemblages^61^. The argillic index shows a broadly distributed NE–SW trending zone centered on G. Umm Salatit and extending toward G. Al Hamri, closely following the mapped ophiolite–island arc contact and the principal shear corridor. Ferrous silicates (Fig. 4b), chlorite, biotite, actinolite, and hornblende, are detected by Fe²⁺ absorptions near 1.0–1.6 μm and concentrated southeast of G. Umm Salim, reflecting mafic to ultramafic lithologies or greenschist-facies metamorphic assemblages in the ophiolite sequence. Propylitic alteration (Fig. 4c) is the most areally extensive alteration type, mapped by diagnostic absorptions of chlorite (Fe-OH near 2.25 μm), epidote (Al-OH near 2.33 μm), and calcite (CO₃ near 2.33–2.35 μm). The propylitic halo forms the outermost, lowest-temperature envelope of the hydrothermal system and covers much of the G. Umm Salatit-G. Umm Salim belt, consistent with pervasive greenschist-facies metamorphism and regional carbonate alteration that characterize ophiolitic terranes in the Central Eastern Desert^62,63^. Ferrugenation (Fig. 4d) zones enriched in goethite, hematite, and limonite are mapped through Fe³⁺ charge-transfer and crystal-field absorptions in the VNIR (0.48–0.90 μm). These zones are concentrated around G. Al Hamri in the northern sector and along discrete NE–SW aligned corridors, suggesting oxidative weathering of sulfide-bearing rocks. Such gossanous caps are recognized as geochemical pathfinders for underlying primary sulfide mineralization in the Eastern Desert gold province^62,63^. Hydroxyl-bearing minerals (Fig. 4e) show limited but spatially focused expression in the southeastern sector, indicating narrow zones of intense phyllic or advanced argillic alteration associated with localized fluid conduits.

#### Spatial relationship to lithology and structure

The composite alteration map (Fig. 4f) reveals a systematic spatial zonation. Propylitic alteration (yellow) dominates the ophiolite–metavolcanic belt, grading inward to argillic (blue) along the principal shear zones. Ferrugenation (magenta) caps structurally elevated blocks and is most intense near granite–ophiolite contacts, a pattern that mirrors the oxidation front above sulfide-bearing shear zones. This alteration architecture is consistent with classical orogenic gold hydrothermal models in the Arabian–Nubian Shield, where structurally controlled, CO₂-bearing aqueous fluids migrate upward along suture-parallel shear zones, depositing gold in quartz–carbonate veins within narrow alteration envelopes^63–65^. Critically, the Au occurrence at G. Umm Salatit (Fig. 1d) falls within the zone of overlapping argillic, propylitic, and ferrugenation signatures, confirming the spatial coincidence of multi-style alteration with known mineralization. Comparison with previous alteration mapping studies using ASTER data in the Central Eastern Desert^62,66^ reveals that EnMAP provides finer mineralogical discrimination owing to its continuous SWIR spectral coverage, which resolves the subtle spectral shifts between kaolinite (Al-OH at ~ 2.17 μm), illite (~ 2.20 μm), and muscovite (~ 2.22 μm) that fall within a single ASTER band. This enhanced resolution is particularly valuable for distinguishing argillic from phyllic alteration, a distinction with direct exploration significance as phyllic zones typically lie proximal to mineralized veins.

### Surface lineament extraction from Sentinel-1 SAR

**Fig. 5:**
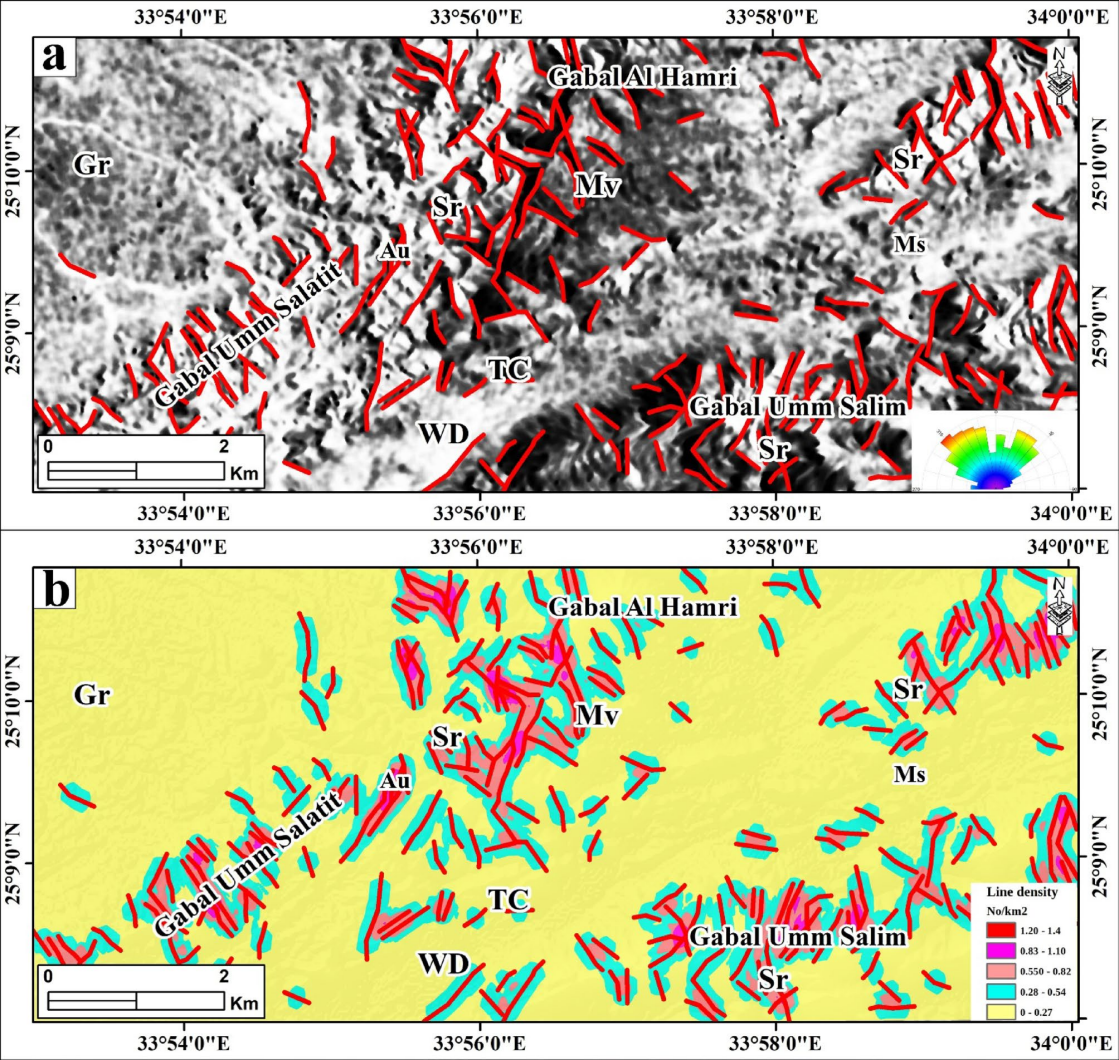
(**a**) Lineaments extraction and rose diagrams based on Sentinel-1 data. (**b**) Lineament density map of Sentinel-1 data. Created by ArcGIS Desktop 10.8. software; https://www.esri.com/en-us/arcgis/products/arcgis-desktop/overview, ENVI v. 5.3. software; https://www.l3harrisgeospatial.com/Software-Technology/ENVI, Geomatica PCI software, and RCOKWORK v. 18 software; https://www.rockware.com/product/rockworks/.

In this study, we used edge-detection techniques and directional filtering on the C-band backscatter data to automatically extract surface lineaments from Sentinel-1 SAR images (Fig. 5a). SAR data are naturally sensitive to rough surfaces and small changes in the topography, which makes it possible to find fractures, faults, and lithological contacts that may not be obvious in optical images. The recovered lineaments show a dense network of structural features with sets that are mostly NE–SW, NW–SE, N–S, and E–W, as shown in the rose diagram (Fig. 5a). The main NE–SW lineament trend runs parallel to the Najd fault system and the Barramiya–Um Salatit shear belt, which is one of the most important gold-controlling structures in the Central Eastern Desert^62^. The lineament density map (Fig. 5b) shows how many structural characteristics there are in a certain area (No/km²). The areas with the most density (1.20–1.40 No/km²) are all along the G. Umm Salatit–G. Al Hamri axis, which is exactly where the ophiolite–island arc contact and the main NE–SW shear corridor meet. Secondary density maxima can be found along the NW–SE cross-cutting structures close to G. Umm Salim. The western and eastern margins of syn-orogenic granites (Gr) and the southern lowlands of wadi deposits (WD) have the lowest lineament density (0–0.27 No/km²). This is because the granitic plutons and the sedimentary fill of the wadi system are relatively massive and undeformed.

The fact that high lineament density and argillic–propylitic alteration anomalies occur in the same area indicates that structure controls the movement of hydrothermal fluids. Shear zones and the fracture networks that go with them acted as high-permeability pathways that moved metamorphic and magmatic fluids across the ophiolite–island arc assemblage. This caused the alteration zonation that we see. This coupling of structural changes is typical of orogenic gold systems worldwide. It has been well-documented in the ENE–WSW Barramiya–Um Salatit shear belt, where gold-quartz veins are found at structural intersections and dilational sparks within transpressional shear zones.

### Aeromagnetic data

Areas with complex geology commonly exhibit higher mineralization potential, although the degree of association varies with deposit type and geological setting^67^. A clear example is the Central Eastern Desert of Egypt (Fig. 1d). Intense tectonic deformation within the basement complex of the Central Eastern Desert formed pathways for mineral-rich fluids and magmas to move through the crust. Gabal Um Salim, in the Central Eastern Desert of Egypt, occupies the northeastern part of the ENE-trending Barramiya–Um Salatit ophiolitic belt and is characterized by ophiolitic serpentinite (SR), metavolcanics (MS), serpentine–talc–carbonate rocks (TC), volcaniclastic metasediments (Ms), syn-orogenic granites (Gr), and wadi deposits (WD)^68^. Gold is chiefly hosted by narrow quartz, carbonate veins and veinlets, and as minor disseminations along shear zones that cut the serpentinized ultramafic rocks and adjacent metavolcanics, i.e., a classic shear-hosted setting in ophiolitic rocks^3,40^.

**Fig. 6:**
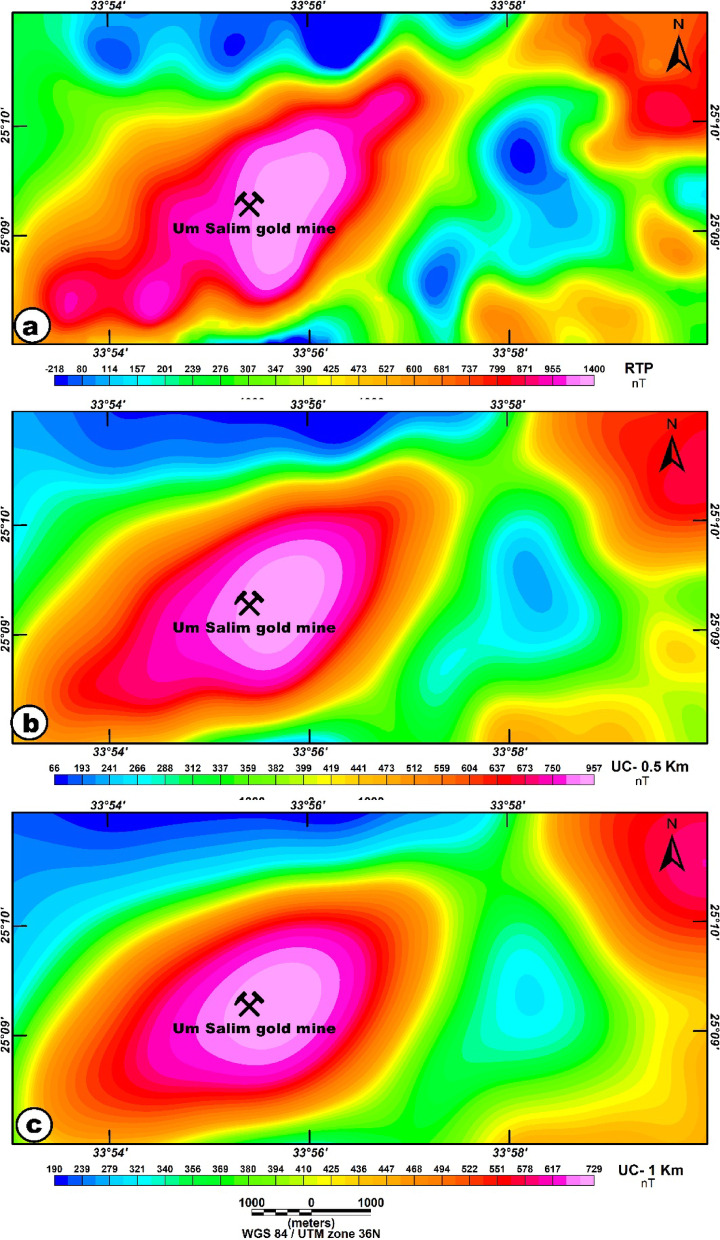
Magnetic maps of the Um Salim area. (**a**) Reduced-to-Pole (RTP) magnetic anomaly map. (**b**) Upward continued (UC) RTP map at 0.5 km. (**c**) UC-RTP map at 1 km. The figure was generated using Geosoft Oasis montaj (v8.3.3) software (Seequent; https://www.seequent.com/products-solutions/oasis-montaj/).

The RTP map (Fig. 6a) shows magnetic anomalies of both positive and negative polarity, which are attributed to the diverse rock types present. The magnetic anomaly readings range from a low of -218 nT to a high of 1400 nT. The strength of a magnetic anomaly is controlled by the magnetization of the underlying rocks, which in turn reflects their magnetic susceptibility at a given location^69^. A well-defined, positive magnetic response characterized by an amplitude of approximately 1400 nT is detected directly above the Au gold mine (Um Salim gold mine). This anomaly is most likely related to magnetite-bearing lithologies and structural features that provided favorable pathways for the movement and accumulation of hydrothermal fluids. The close spatial association between magnetic highs and gold occurrences in this region emphasizes the structural control on mineralization within the study area. Similar relationships have been documented in other gold provinces worldwide, where magnetic anomalies frequently serve as reliable indicators of ore localization and exploration targets^70,71^. This suggests that the identified anomaly not only marks the current deposit but may also guide further exploration in adjacent areas with comparable geophysical signatures. The elongated positive magnetic anomaly is commonly linked to metavolcanics and serpentinized ultramafic rocks. These lithologies typically display strong magnetic susceptibilities due to their mineral composition^72^. In contrast, wadi deposits and older granitoid bodies exhibit relatively low magnetic responses, as illustrated in Fig. 6a.

Figure 6b and c show the upward continuation of the RTP magnetic map at 0.5 km and 1 km, respectively. This approach is applied to provide clearer insight into the basement’s depth distribution and its dominant structural orientations. The equivalent source depths are estimated to be approximately 0.25 km and 0.5 km, which corresponds to the established assumption that upward continuation to a level z corresponds to geological sources located approximately z/2 below the surface^47,73^. The elongated, high-amplitude positive magnetic anomaly observed in the study area persists with minimal attenuation for up to 1 km, indicating a deeply rooted source, evidently of plutonic origin. Nonetheless, qualitatively interpreting RTP and upward continued maps can be unclear, ambiguous, and sometimes uncertain^74,75^. Signal-processing techniques are recommended to enhance the precision of interpretation. Among these, edge-detection filters are particularly valuable in mineral exploration because they highlight anomaly boundaries and help delineate magnetic sources and structural elements, including fractures, faults, and lithological contacts, which strongly influence mineral distribution^17^.

**Fig. 7:**
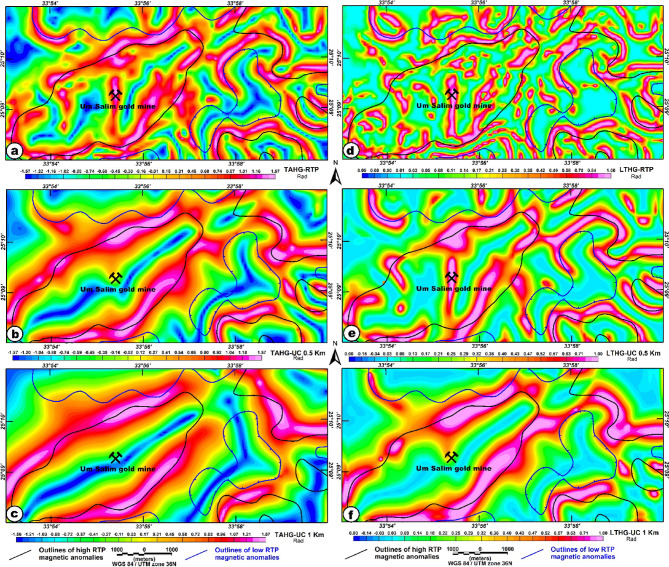
(**a**) TAHG-RTP; (**b**) TAHG-UC at 0.5 km; (**c**) TAHG-UC at 1 km; (**d**) ILTHG-RTP; (**e**) ILTHG-UC at 0.5 km; (**f**) ILTHG-UC at 1 km of the investigated area. The figure was generated using Geosoft Oasis montaj (v8.3.3) software (Seequent; https://www.seequent.com/products-solutions/oasis-montaj/).

To improve the accuracy of structural mapping, two advanced edge-detection filters, TAHG and ILTHG, were applied to the RTP and upward-continued datasets. These filters outperform conventional techniques by producing high-resolution images that precisely delineate the boundaries of magnetic sources at both shallow and greater depths^52^. The effectiveness of TAHG and ILTHG in enhancing subtle variations enables the clear distinction of anomalies associated with both weak and strong magnetic responses, a task where traditional RTP maps often fall short, particularly in the southeastern sector of the study area (Fig. 7). This refinement is of considerable significance for mineral exploration, as the detection of weak or concealed magnetic anomalies may evidence hidden mineralization zones or reveal structural pathways that have guided hydrothermal fluid flow. Recent studies have demonstrated that advanced edge-detection filters provide robust tools for interpreting complex geophysical datasets and identifying geological features that directly influence the distribution of mineral deposits^17,47^.

**Fig. 8:**
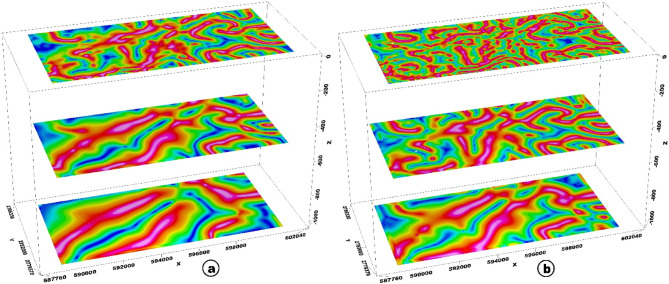
3D plane view of (**a**) TAHG-RTP, TAHG-UC at 0.5 km, and TAHG-UC at 1 km, (**b**) ILTHG-RTP, ILTHG-UC at 0.5 km, and ILTHG-UC at 1 km in the study area. The figure was generated using Geosoft Oasis montaj (v8.3.3) software (Seequent; https://www.seequent.com/products-solutions/oasis-montaj/).

The outcomes of applying TAHG and ILTHG to the RTP dataset are shown in Fig. 7, where the filters successfully define the boundaries of magnetic sources and structural trends across the area. These methods yield highly detailed and reliable outputs, offering comprehensive insight into the subsurface framework. These filters sharpen peak responses at source margins, thereby enhancing the resolution of both strong and subtle magnetic anomalies^52,76,77^. The scaling parameter α in the ILTHG filter is a positive factor determined by the interpreter. Previous testing by^52^ demonstrated that optimal results are typically obtained when α ranges between 2 and 10. In this study, α = 2 was selected after comparative testing, yielding enhanced boundary sharpness while minimizing noise amplification and preserving structural continuity. The main anomaly trends follow ENE–WSW, NE–SW, and NW–SE orientations, with a subordinate N–S set also observed (Fig. 7), consistent with regional tectonic fabrics that control the formation of ore/minerals in the Central Eastern Desert^17^. When applied to upward-continued datasets at 0.5 km and 1 km (Fig. 7b,c,e,f), the number of anomalies decreases noticeably, indicating that many of the identified features represent relatively shallow sources. This depth-dependent behavior is further demonstrated in the TAHG and ILTHG maps (Fig. 8a and b), which provide three-dimensional plan views of magnetic variations at different continuation heights. Together, these results highlight the efficiency of advanced filtering techniques in resolving structural controls that govern mineralization, a finding consistent with similar applications in other gold-bearing provinces worldwide^78,79^. The use of both TAHG and ILTHG in this study is motivated by their different responses to the shape and amplitude of magnetic gradients, which allows them to highlight complementary aspects of the signal^75,80^. The TAHG operator, based on the tilt angle of the total horizontal gradient, enhances the total amplitude of lateral gradients and is particularly effective for delineating broad, high-contrast boundaries and continuous structures, such as major lithological contacts and the overall geometry of large intrusive bodies^81^. In contrast, logistic-based filters such as ILTHG normalize the total horizontal gradient using a logistic function, which reduces the dominance of very strong anomalies and amplifies subtle, rapidly varying gradients; this behavior makes ILTHG better suited to resolving closely spaced or low-contrast features such as minor faults, fracture swarms, and narrow alteration zones that may be blurred in TAHG results^75,80^. In our dataset, TAHG therefore provides a robust first-order framework for the main structural blocks, whereas ILTHG refines this picture by revealing finer-scale discontinuities within and along these blocks; where both operators delineate the same trends, their agreement increases confidence in the interpreted structures, and where ILTHG adds detail, it augments rather than contradicts the TAHG-derived pattern^75,82^.

**Fig. 9:**
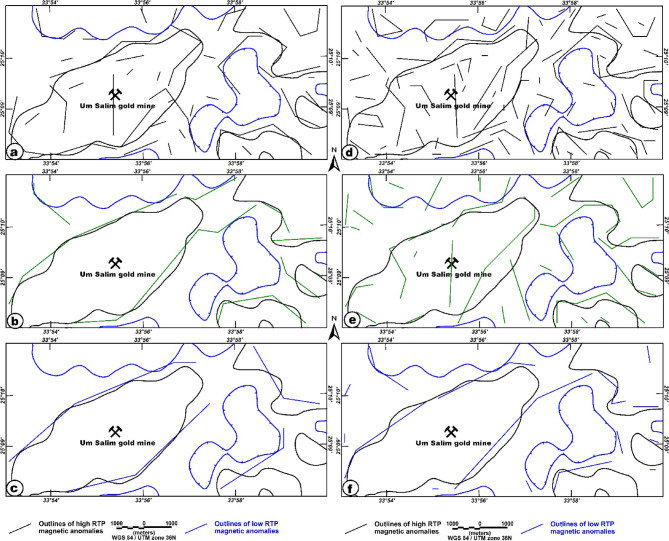
CET structural maps of (**a**) TAHG-RTP; (**b**) TAHG-UC at 0.5 km; (**c**) TAHG-UC at 1 km; (**d**) TAHG-RTP; (**e**) TAHG-UC at 0.5 km; (**f**) TAHG-UC at 1 km of the study area. The figure was generated using Geosoft Oasis montaj (v8.3.3) software (Seequent; https://www.seequent.com/products-solutions/oasis-montaj/).

**Fig. 10:**
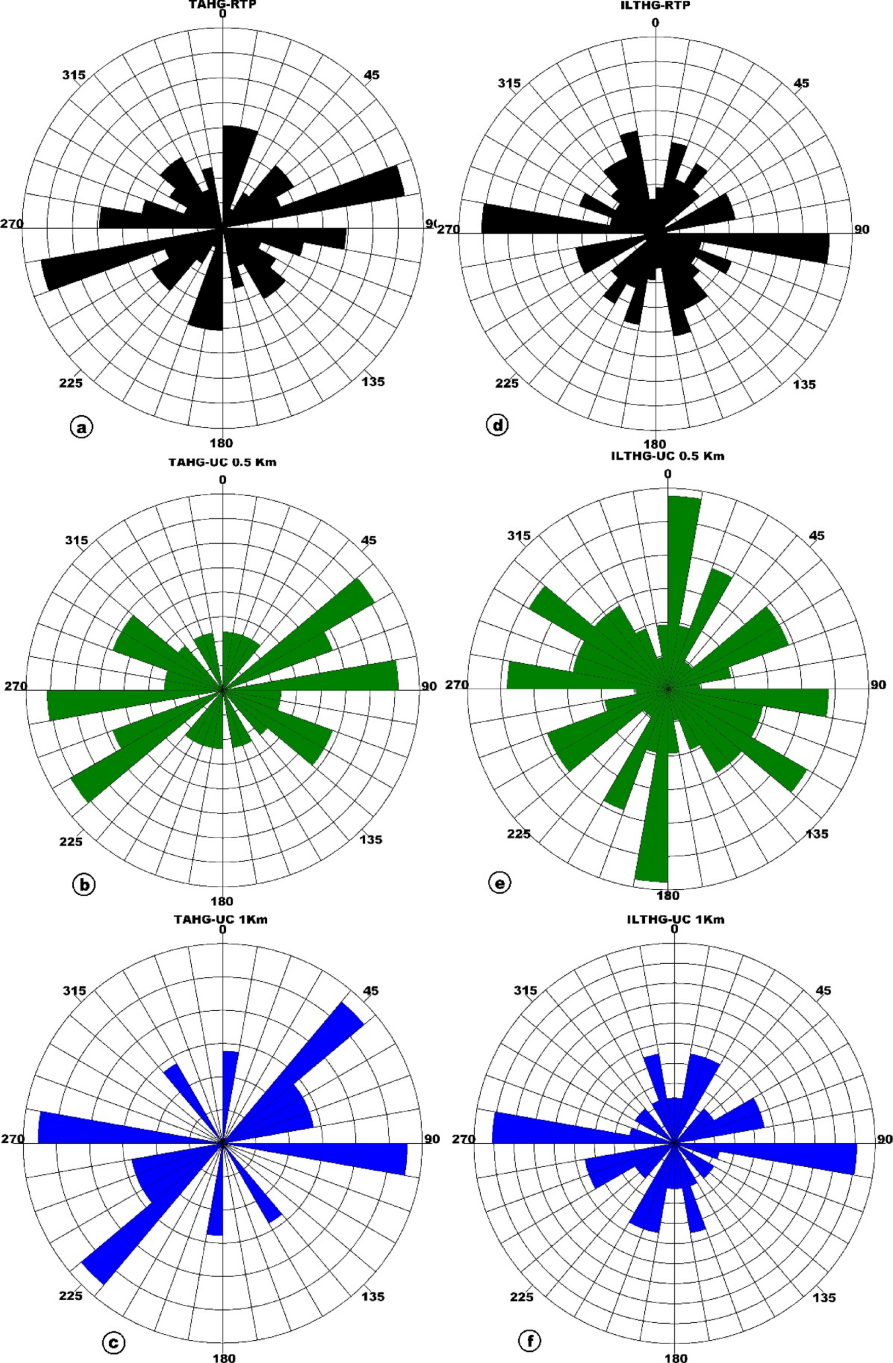
Rose diagrams of CET-generated structural maps from (**a**) TAHG-RTP; (**b**) TAHG-UC at 0.5 km; (**c**) TAHG-UC at 1 km; (**d**) ILTHG-RTP; (**e**) ILTHG-UC at 0.5 km; (**f**) ILTHG-UC at 1 km of the study area. The figure was created using RockWare (2017) (https://www.rockware.com/product/rockworks).

Figure 9 illustrates CET structural maps constructed from edge-enhanced analyses of RTP data, which were upward continued to 0.5 km and 1 km and processed using TAHG and ILTHG filters. This builds on the insights from Fig. 7, offering a more refined view of subsurface structures relevant to early-stage mineral exploration. Analysis of these maps (Fig. 9) alongside the rose diagrams (Fig. 10) reveals that applying TAHG and ILTHG to the highlighted dominant structural orientations, mainly ENE–WSW, NE–SW, and NW–SE, with a subordinate N–S trend, is effective. The structural architecture delineated by the magnetic filtering highlights a complex tectonic imprint within the Central Eastern Desert^83,84^. The NE–SW and NW–SE orientations derived from variance- and texture-based correlations are not random features; they represent distinct deformation episodes within the region’s tectonic history. The NE–SW set corresponds to the early D1 phase, while the NW–SE lineaments reflect the subsequent D2 phase, as recognized in previous structural studies^62,65,85^. Linking these orientations to established deformation phases provides a reliable framework for understanding regional tectonic development and assessing its control on mineralization processes. Two major deformation episodes mark the tectonic history of the area. The first (D1), produced by NNW–SSE compression, is expressed by NE–SW structural fabrics that are well developed in the deformed ophiolitic blocks and accommodated along the dextral Baramiya–Mubarak shear zone. A subsequent phase (D2), associated with NNE–SSW shortening, generated the NW–SE structural grain that presently outlines the principal lithological boundaries. Within the ophiolitic units and mélange matrix, these deformation stages are further emphasized by low second-moment values and elevated dissimilarity, reflecting the intense shearing and structural complexity characteristic of this polyphase tectonic evolution. Collectively, the interplay between D1 and D2 illustrates the sequential tectonic processes that shaped the region’s architecture and exerted a strong control on its mineralization potential.

Moreover, integrating textural correlation with structural orientation reveals the overprinting relationships between the two phases. Sub-horizontal trends, ranging from ENE–WSW to WNW–ESE, are a key structural element in the investigated area. These orientations are best interpreted as the combined imprint of D1- and D2-related shortening, where successive phases of deformation interacted to generate a composite fabric. This superposition not only underscores the complexity of the tectonic evolution but also provides important clues for understanding the structural controls on mineralization pathways. The emergence of sub-vertical N–S lineaments, especially where they intersect and offset earlier fabrics in a sinistral sense, is attributed to the younger D3 phase. This deformation stage, associated with E–W oblique convergence, introduced a new structural grain that overprinted earlier trends, further complicating the region’s tectonic framework^62^. Lineament analysis provides a practical framework for mineral exploration by highlighting structural corridors that control fluid flow and ore deposition. In many gold-bearing terranes, major shear zones, fractures, and fault intersections commonly serve as conduits for hydrothermal fluid flow, concentrating mineralization along structurally prepared zones. Global examples include the Ashanti Belt in Ghana, where gold mineralization is closely associated with NE-SW trending shear zones^86^, and the Kalgoorlie Terrane in Western Australia, where the Boulder-Lefroy Fault Zone hosts world-class deposits such as the Super Pit^87^. In Canada’s Abitibi Greenstone Belt, the Destor-Porcupine and Larder Lake–Cadillac fault zones are major conduits for gold-bearing fluids, with significant deposits, such as Hollinger and Kerr-Addison, located along these structures^88^.

**Fig. 11:**
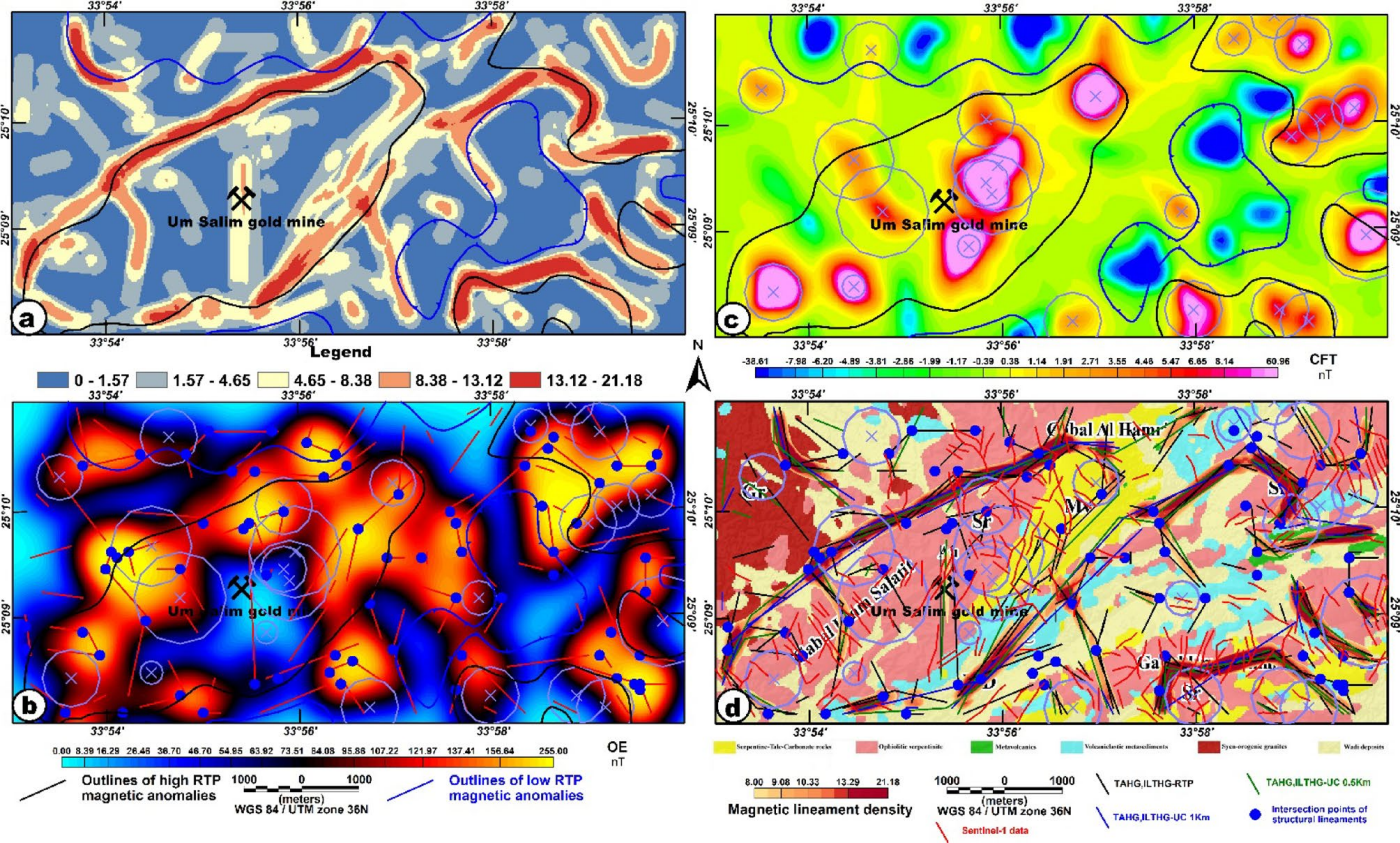
(**a**) Magnetic Lineament Density map, (**b**) Entropy heat map; blue dots show intersection points of structural lineaments derived from integrated edge-detection analysis, (**c**) Circular Feature Transform (CFT). (**d**) composite map superimposing the main geophysical results (magnetic lineaments, high magnetic lineament density, circular anomalies) on the geological map of the study area. The figure was created by ArcGIS Desktop 10.8. (https://www.esri.com/enus/arcgis/products/arcgis-desktop/overview/), and Geosoft Oasis montaj (v8.3.3) software (Seequent; https://www.seequent.com/products-solutions/oasis-montaj/).

The CET grid analysis reveals structural complexity and linear magnetic anomalies within the Um Salim region, as illustrated in Fig. 9. This interpretation is corroborated by the structural lineament density map (Fig. 11a), which delineates a network of magnetic discontinuities. These features correspond to dykes, lithological contacts, faults, and shear zones, with dominant orientations in the ENE-WSW, NE–SW, NW–SE, and N–S directions. Notably, elevated lineament densities are observed along prominent fault systems. The lineament density of Fig. 11a was categorized into three zones: (i) high to very high (8.38–21.18 m/km²), (ii) intermediate (1.57–8.38 m/km²), and (iii) low (0–1.57 m/km²). The high to very high lineament density highlighted zones of intense structural disruption. A dense network of faults, fractures, or shear zones characterizes these areas. Moderate lineament density, suggesting localized fracturing or faulting. These areas may have experienced some degree of tectonic activity and could host structurally controlled alteration zones. In contrast, low lineament density indicates relatively intact or undeformed bedrock. These regions experienced minimal tectonic disturbance and are less likely to serve as pathways for hydrothermal fluids.

The Um Salim gold mine is in a yellow-to-orange zone (4.65–13.12 m/km²), adjacent to or partially overlapping areas with higher density values to the east and southeast. These high-density zones reflect major fault corridors or shear zones that may have served as conduits for hydrothermal fluids, thereby enhancing mineralization potential. The linear red and orange bands exhibit prominent structural trends, primarily oriented ENE-WSW, NW–SE, and NE–SW. These orientations align with known regional fault systems and could represent reactivated Precambrian shear zones. The eastern and southeastern sectors of the map display continuous high-density lineaments, making them strong candidates for further mineral exploration. The western and southwestern edges also show prominent red bands, potentially indicating parallel or intersecting fault systems. Areas with intersections between multiple high-density trends are exciting, as these structural intersections often serve as favorable sites for ore deposition. Areas with a high density of lineaments show a clear spatial association with regions of increased magnetic amplitude, as observed in the RTP, TAHG, and ILTHG maps. Lineaments play a critical role in mineralization by facilitating the ascent and localization of mineral-bearing hydrothermal fluids within the upper crust. Their structural control on fluid flow makes them reliable proxies in mineral exploration, as supported by numerous recent studies^20,77,89,90^. Accordingly, zones with high lineament density are often prioritized as high-potential targets for mineral resource development.

The area’s structural framework is overlaid on the orientation entropy heat map (OE) (Fig. 11b). The map delineates geological lineaments of varying lengths, several of which extend into or link with adjacent lineaments elsewhere in the study area. The structural framework of the area is overlaid on the orientation entropy heat map. As shown in Fig. 11b, the OE distribution highlights zones of high orientation variability among lineaments, indicating areas of high structural complexity. These zones of high structural complexity (8.38–21.18 m/km²) contrast with areas of low complexity (0–1.57 m/km²). Notably, the known gold mining sites are clustered near high-complexity zones, indicating a strong spatial association between structural complexity and mineralization. There is a strong correspondence between the orientation-entropy (OE) heat map (Fig. 11b) and the structural lineament density map (Fig. 11a). This agreement is expected: high lineament density increases the diversity of azimuths within a neighborhood, raising OE values; conversely, sparse or uniformly oriented lineaments yield low OE. Therefore, the co-location of OE highs with density highs validates that both metrics consistently capture zones of intensive, multi-directional fracturing, key structural complexity indicators. Also, the structural interpretation maps (Fig. 10) were enhanced by superimposing contour outlines of the RTP magnetic anomalies to illustrate the spatial relationships between the anomaly sources and the extracted structural features. This integrated visualization approach enables comparison among magnetic highs/lows, mapped lineaments, density concentrations, and circular features, thereby improving interpretation of source and structure correspondence and reinforcing the geological consistency of the results.

Reference^91^ note that magnetic surveys can aid mineral targeting when hydrothermal alteration from the mineralizing event modifies the host rock’s magnetic properties, thereby enhancing detectability. Accordingly, the circular anomalies detected by CET-PA are interpreted here as probable porphyritic intrusions, because their geometry is consistent with the expected morphologic expression of porphyry centers and their associated alteration shells^60^. In porphyry systems, hydrothermal alteration commonly forms near-circular, concentric halos that encircle an approximately circular central intrusion^91^. The intrusion and its immediate alteration halo commonly produce a magnetic signature characterized by a central positive anomaly (magnetic high) encircled by a comparatively subdued, annular low (Fig. 11c). Alteration modifies the magnetic characteristics of host rocks, yielding distinctive anomalies in aeromagnetic data^69^. Figure 11c shows that porphyry intrusions and their surrounding alteration zones are commonly associated with increased magnetic responses, whereas more distant alteration areas exhibit low magnetic signatures^91^. Depending on the nature of the hydrothermal processes involved, magnetite can either form or be destroyed, resulting in corresponding positive or negative magnetic anomalies^58^. Dyke-like porphyry features are predominantly found within sheared ultramafic units (Fig. 11b,c)^3,92^. These features often align with structurally complex zones, where multiple trends intersect. Regions with elevated structural complexity exhibit a higher concentration of porphyry features (Fig. 11b), suggesting a greater potential for additional ore deposition. Additionally, existing gold mining sites are spatially associated with the identified porphyry features, often occurring directly above or in close proximity to them (Fig. 11b,c). This spatial correlation reinforces the genetic link between porphyry intrusions and gold mineralization, highlighting their significance in targeting exploration zones. The composite overlays (Fig. 11d) demonstrate a clear spatial agreement between magnetically derived lineaments, high-density structural zones, and mapped geological contacts. Major magnetic trends coincide with known fault systems, while circular anomalies align with intrusive and alteration-related units. This structural convergence strengthens the geological validity of the aeromagnetic interpretation and enhances confidence in the identified target zones.

**Fig. 12:**
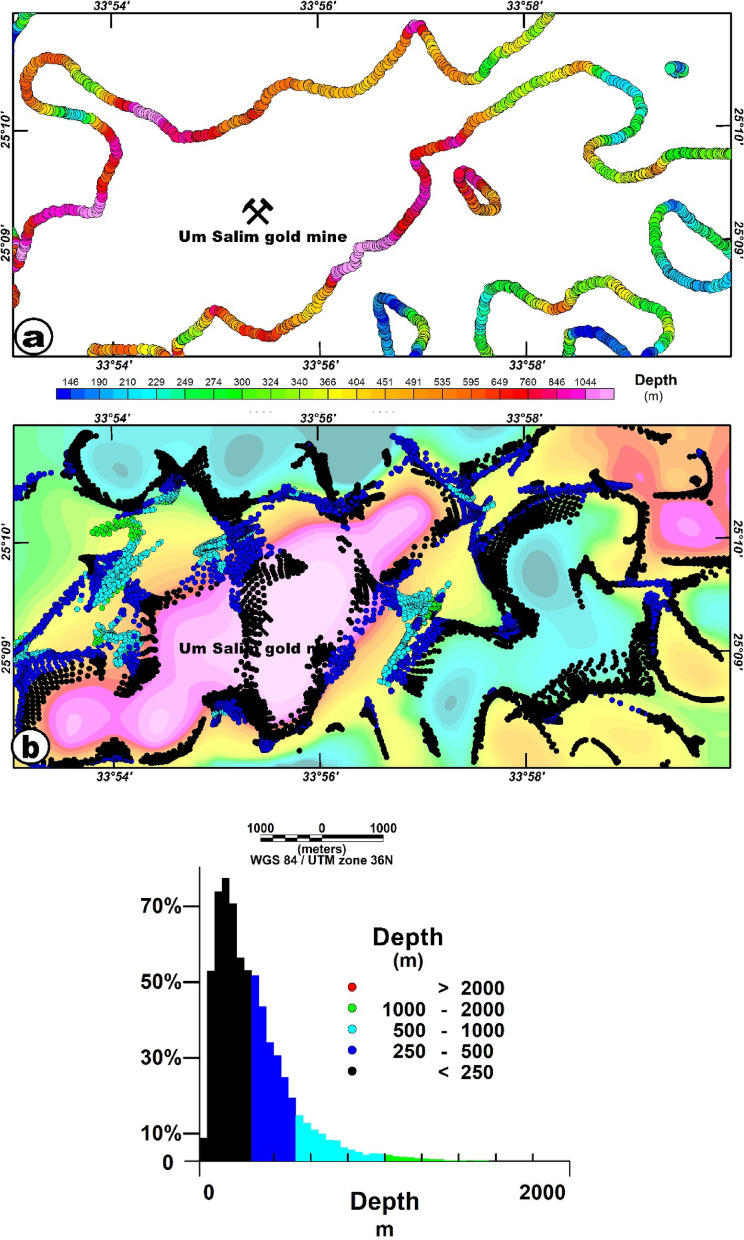
Depth solutions (**a**) (TD) (**b**) EUD methods.Geosoft Oasis montaj (v8.3.3) software (Seequent; https://www.seequent.com/products-solutions/oasis-montaj/).

Accurate delineation of the depth and geometry of buried structures is crucial for understanding the controls on mineralization. Recent studies have demonstrated that combining quantitative depth-to-source estimation with edge-enhancement filters can substantially sharpen the imaging of subsurface architectures^93^. Motivated by this, we applied both Euler Deconvolution (EUD) and Tilt-depth method (TA) to the RTP aeromagnetic data to derive robust depth estimates and highlight structurally controlled features potentially associated with hydrothermal alteration (Fig. 12). The integration of these analytical approaches provides robust and high-resolution depth estimations, offering a stronger foundation for interpreting subsurface geology (Fig. 12). Their application is particularly valuable because they enable the spatial delineation of both isolated and clustered magnetic sources, a capability well established in geophysical studies^94^. The EUD technique was implemented using a structural index of 0, enabling accurate recognition and separation of the identified structural elements^47^. The resulting solution distributions reveal that magnetic sources predominantly follow ENE-WSW, NE–SW, NW–SE, and N–S directions (Fig. 12b). These findings further validate, and are in agreement with, the results obtained from advanced edge-enhancement techniques. To further interpret subsurface architecture, statistical assessments of the derived depths were conducted, highlighting that most magnetic sources occur within the upper 0–1000 m, indicating relatively shallow bodies. However, depths of approximately 1500 m point to the presence of more deeply rooted dykes and fault-controlled structures (Fig. 12b). The application of the Tilt Depth (TD) technique implies that magnetic bodies occur at depths ranging from the surface to approximately 1000 m. The results show that the majority of solutions fall within the upper 1000 m, reflecting that most structures are concentrated within the shallow crust (Fig. 12a). When combined with aeromagnetic interpretation, the EUD and TD approaches significantly improved the geological understanding of the area, allowing more accurate delineation of major structural elements such as shear zones, dykes, and fault systems. This improved structural definition provides valuable insights into features that act as conduits for hydrothermal fluid circulation.

Collectively, these findings demonstrate the effectiveness of the applied methodology in characterizing structural trends and depth variations across the study area. A previous fieldwork study by the Egyptian Geological Survey in the 1970s^95^ documents Um Salim as a vein-hosted gold occurrence developed within sheared ultramafic rocks. Regionally, the Al-Barramiya–Um Salim belt encompasses extensive ultramafic units [3], which provide favorable hosts for gold, particularly where quartz veins intersect or crosscut these rocks^96–98^. Previous work also shows that serpentinite rocks can host gold at concentrations of roughly ~ 200 ppb^93,99^. Our results are consistent with regional studies that link structural complexity, magnetic contrasts, and gold occurrence, reinforcing the validity of our targeting criteria. This cross-study agreement reduces uncertainty and supports prioritizing structurally complex zones^18,96,99–102^.

This study demonstrates that integrating advanced magnetic filtering, structural analysis, and depth-to-source modeling provides a robust and reliable framework for identifying structurally controlled gold targets in ophiolitic terrains. The combination of TAHG/ILTHG filters, CET-based structural metrics, lineament density and entropy mapping, and EUD/TD depth estimation reveals a multi-phase tectonic architecture in which hydrothermal fluids were focused along reactivated shear corridors, fracture intersections, and porphyry-linked intrusive pathways. The strong spatial correspondence between structural complexity zones, magnetic anomalies, and known gold occurrences validates the methodology. It highlights several additional high-priority exploration targets, particularly in the eastern and southeastern sectors of the Um Salim region. Overall, this integrated geophysical–structural approach not only refines understanding of the subsurface architecture but also provides a robust, transferable exploration model applicable to similar gold-bearing belts worldwide. It is important to emphasize that the aeromagnetic interpretation is inherently non-unique, as different magnetization distributions can produce similar anomalies^103^. Moreover, the legacy survey’s line spacing and flight altitude constrain resolution and depth accuracy, so inferred depths and fault geometries represent first-order estimates. Consequently, the structurally focused magnetic targets require validation through detailed field mapping or drilling data to refine the interpretation. Such validation would refine the structural framework proposed here and provide new opportunities for targeted exploration. To overcome these constraints, the study implements an integrated interpretation strategy that jointly utilizes EnMAP hyperspectral imagery, Sentinel-1 SAR–derived lineaments, and aeromagnetic data. Each dataset independently constrains surface lithology, alteration patterns, and structurally deformed zones, thereby reducing magnetic ambiguity and enhancing the reliability of the resulting geological model.

### Datasets integration

The integrated analysis of EnMAP hyperspectral data, Sentinel-1 SAR lineaments, and aeromagnetic datasets reveals a coherent, multi-scale architecture that tightly controls gold mineralization in the Um Salim area. EnMAP SVM–MNF classification provides a high-fidelity lithological framework in which the ophiolitic serpentinite belt, talc–carbonate pods, metavolcanics, and syn-orogenic granites are clearly resolved, with overall accuracies exceeding 95% and geologically consistent contact geometries. Within this spectral framework, targeted hydrothermal alteration indices delineate a zoned system in which a broad propylitic halo blankets the ophiolitic serpentinite (SR) and serpentine–talc–carbonate (TC) units, grading inward to structurally focused argillic, ferruginous, and silicified zones that envelop the main NE–SW shear corridor, the four alteration types most diagnostic of orogenic gold mineralization. Critically, the known Au occurrence (Fig. 1d) is spatially restricted to the highly magnetic and structurally deformed zone interpreted as the Barramiya–Um Salatit shear belt, where aeromagnetic inversion has independently confirmed the presence of elongated ENE–WSW magnetic bodies consistent with serpentinized ultramafic host rocks, in agreement with classical orogenic gold models in the Arabian–Nubian Shield^62–65^.

The structural framework of the Um Salim area is consistently delineated across three independent datasets, reinforcing the reliability of the interpreted lineament network and directly addressing the need for cross-dataset structural correlation. Geological mapping reveals dominant NE–SW and NW–SE structural trends expressed as shear zones, mylonitic foliations, and dyke-like porphyry intrusions, reflecting multi-phase deformation (D1–D3) superimposed on the ophiolitic and island-arc assemblages. These surface structures are independently reproduced by Sentinel-1 SAR-derived lineaments, which resolve the same NE–SW, NW–SE, N–S, and E–W trends through automated edge-detection and the LINE algorithm applied to C-band backscatter data. Aeromagnetic edge-detection filters (TAHG and ILTHG) further delineate subsurface structural boundaries that mirror both the geological mapping and SAR lineament orientations, confirming that the mapped fault and shear systems are deeply rooted rather than superficial features (Figs. 9 and 11). This three-way structural agreement between geological mapping (Fig. 1d), Sentinel-1 SAR lineaments (Fig. 5a), and aeromagnetic fabrics provides robust (Fig. 8), multi-dataset validation of the interpreted deformation architecture. It eliminates interpretational ambiguity that would arise from any single dataset alone.

Sentinel-1 SAR lineament extraction and density–entropy analysis reveal a polyphase structural architecture dominated by ENE–WSW, NE–SW, NW–SE, and subordinate N–S trends. High lineament density and elevated orientation entropy define structurally complex corridors that spatially overlap alteration halos and gold occurrences. This structural convergence mirrors global shear-hosted gold systems such as the Ashanti Belt (Ghana), the Kalgoorlie Terrane (Australia), and the Abitibi Greenstone Belt (Canada), where mineralization preferentially localizes at fault intersections and reactivated shear zones^104,105^. Aeromagnetic interpretation adds the critical subsurface dimension: RTP data delineate a persistent ~ 1400 nT anomaly centered on the Um Salim mine, attributed to magnetite-bearing metavolcanics and ophiolitic serpentinite, while upward continuation confirms its deep-seated nature. Advanced edge-detection (TAHG, ILTHG) and CET structural analysis extract ENE–WSW, NE–SW, and NW–SE magnetic fabrics that mirror the SAR-derived fracture patterns and are directly linked to the sequential D1–D2–D3 deformation history. Lineament density and orientation entropy maps pinpoint zones of maximum structural complexity where alteration anomalies, magnetic highs, and known gold occurrences converge. Circular magnetic features revealed by the CFT, spatially associated with sheared ultramafic units and high-complexity nodes, reinforce a hybrid structural–intrusion-related control on mineralization. Depth solutions from Euler Deconvolution and Tilt-Depth modeling indicate that most magnetic sources occur within 0–1000 m, with deeper (~ 1500 m) bodies representing intrusive conduits that likely focused hydrothermal fluid ascent.

The strong spatial correspondence between alteration anomalies, structural density maxima, magnetic gradients, and depth solutions significantly reduces interpretational ambiguity and enhances geological confidence. Importantly, the agreement between independent spectral, radar, and magnetic datasets acts as mutual validation, strengthening the robustness of the targeting criteria. This integrated workflow demonstrates that high-resolution remote sensing, combined with advanced magnetic filtering and quantitative depth modeling, provides a transferable, scalable exploration strategy for structurally controlled gold systems in Precambrian terranes worldwide.

## Conclusion

This study demonstrates that integrating EnMAP hyperspectral mapping, Sentinel-1 SAR structural analysis, and advanced aeromagnetic processing provides a powerful, multi-sensor framework for delineating structurally controlled gold targets in the Umm Salim segment of the Central Eastern Desert. High-resolution magnetic filters (TAHG, ILTHG), together with MNF-enhanced lithological classifications and SAR-derived lineament density, effectively resolve key structural fabrics and lithological boundaries, revealing dominant ENE–WSW, NE–SW, NW–SE, and N–S lineaments associated with multi-phase deformation (D1–D3) and highlighting the critical role of reactivated shear zones, fracture intersections, and intrusive bodies in focusing mineralization. Depth-to-source solutions from Euler Deconvolution and Tilt-Depth modeling indicate that most magnetic sources are shallow (0–1000 m), whereas deeper bodies (~ 1500 m) likely represent porphyry-related intrusions and fault-controlled conduits for auriferous hydrothermal fluids. Their strong spatial correspondence with multi-style alteration halos and high lineament entropy zones defines several high-priority exploration targets in the eastern and southeastern parts of the study area. It establishes a reproducible, high-resolution targeting strategy applicable to structurally complex gold provinces worldwide.

## Data Availability

The datasets used and/or analyzed during the current study are available from the corresponding author upon reasonable request.
